# Effectiveness of kinesio taping on postoperative morbidity after impacted mandibular third molar surgery: a prospective, randomized, placebo-controlled clinical study

**DOI:** 10.1590/1678-7757-2020-0159

**Published:** 2020-07-13

**Authors:** Ufuk TATLI, Ilke Coskun BENLIDAYI, Fariz SALIMOV, Rengin GUZEL

**Affiliations:** 1 Cukurova University School of Dentistry Department of Oral and Maxillofacial Surgery Adana Turkey Cukurova University, School of Dentistry, Department of Oral and Maxillofacial Surgery, Adana, Turkey.; 2 Cukurova University School of Medicine Department of Physical Medicine and Rehabilitation Adana Turkey Cukurova University, School of Medicine, Department of Physical Medicine and Rehabilitation, Adana, Turkey.

**Keywords:** Impacted tooth, Kinesio taping, Pain, Placebo, Swelling, Trismus

## Abstract

**Objective:**

Our study seeks to investigate the effectiveness of kinesio taping (KT) on postoperative morbidity compared to placebo and control groups after impacted third molar surgery.

**Methodology:**

Sixty patients with impacted mandibular third molar were included in this prospective, randomized, placebo-controlled clinical study. After surgical extraction of the impacted tooth, patients were allocated into three groups (20 patients each): group 1 received KT (kinesio), group 2 received placebo taping (placebo), and group 3 received no taping (control). The groups were compared regarding facial swelling, pain and trismus. Swelling was evaluated using a tape measuring method. Pain was assessed by a visual analog scale and the number of analgesic tablets taken. Trismus was determined by measuring maximum mouth opening.

**Results:**

In the KT group, all parameters reduced significantly on 2^nd^ and 4^th^ postoperative days compared to other groups; however, placebo and control groups revealed comparable outcomes. On 7^th^ day, all groups showed comparable results.

**Conclusions:**

The KT application is an effective method for reducing morbidity after impacted mandibular third molar surgery. However, placebo taping is not as effective as proper taping. Placebo taping shows similar results compared to no taping regarding facial swelling percentage, pain and trismus.

## Introduction

Surgical extraction of impacted third molar teeth is one of the most frequent operations in the field of oral and maxillofacial surgery (OMS), worldwide. Similar to other oral surgical procedures, patients suffer from a number of discomforts and disabilities (swelling, pain, and trismus ongoing seven to ten days after surgery). These morbidities are due to inflammatory response in consequence of surgical trauma. The postsurgical morbidities affecting jaws and face cause significant discomfort to patients. In attempt to reduce inflammatory response associated with surgical trauma and postoperative morbidity after OMS, many adjuvant applications including different surgical techniques,^1^ drugs,^2,3^ drains,^4,5^ low-level-laser therapy,^6,7^ cooling therapy^8^ or physical therapy^9^ have been reported. Drug-related adverse effects, complications and contraindications led researchers to study non-drug methods. One of the adjuvant applications to reduce morbidity after traumatic injuries in sports medicine is kinesio taping (KT), which was introduced as an elastic therapeutic taping technique in 1970s.^10^ The KT technique implements its effect on lymphatic drainage by lifting the skin, thus guiding lymph flow to move from higher to lower pressure sites.^11-13^ Based on this physiologic principle, KT removes congestions of lymphatic fluids and hemorrhages.^11,12^ Taping can also affect mechanoreceptors of joints and muscles, decreasing the nociceptive pain.^13^ On the other hand, a recent meta-analysis concluded that the use of kinesio taping in musculoskeletal disorders had insufficient evidence.^14^

However, despite the vast clinical experience, evidence-based scientific publications on the effectiveness of KT technique in the field of OMS are scarce. Recent studies evaluated the effects of KT technique after OMS procedures including impacted tooth surgery, maxillofacial trauma and orthognathic surgery.^5,15-21^ Recent studies have shown that KT is a useful method for reducing postoperative morbidity by improving blood and lymphatic circulation. However, to the best our knowledge, previous studies comparing the actual effectiveness of KT technique in OMS area did not include a placebo-taping group. Therefore, placebo effect could not be distinguished from the real effectiveness of KT technique in OMS field. Our placebo-controlled clinical study seeks to investigate the actual effectiveness of KT on postoperative morbidity when compared with placebo taping and no taping (control) groups after impacted mandibular third molar surgery. Our hypothesis is that KT reduces postoperative morbidity after surgical extraction of third molar teeth by improving lymphatic drainage; however, placebo taping is not as effective as proper taping.

## Methodology

### Patient Recruitment and Study Groups

This prospective, randomized, placebo-controlled clinical study was performed according to the ethical standards of the Declaration of Helsinki. The study protocol was approved by the institutional ethics committee (No: 26-14-061213). The participants signed an informed consent form after being informed about the study protocol.

A sample size was estimated for the main outcome measure, the pain, considering the results of a previous study^18^ (the required minimum mean difference was set as 1.4 VAS value for statistical significance). To obtain a power of research of 0.80, 54 patients were required (estimated effect size is 0.20), which resulted in 18 patients per group. Considering data loss due to patient dropouts, 10% increase was added in sample size, resulting in 20 patients in each group, totaling 60 patients.

The inclusion criteria for the study were systemic healthiness and presence of impacted mandibular third molars with similar impaction level (Pell and Gregory classification: Class 2, position B).^22^ Exclusion criteria were patients younger than 18 years, pregnant or lactating women, patients with medical contraindications for the surgery, sensitivities to the tape, unwillingness to participate in the study, presence of pericoronitis, odontogenic cyst or tumor associated with impacted tooth.

The patients that met the inclusion criteria were randomly assigned to one of the three study groups using a randomization software (QuickCalcs; GraphPad Software Inc., La Jolla CA, USA). After surgical extraction of impacted mandibular third molar; the patients were assigned into three groups: group 1 received KT (kinesio group), group 2 received placebo taping (placebo group), and group 3 received no taping (control group). The patients had only one tooth extracted and participated in only one treatment group.

### Surgery

All surgeries were performed by the same oral and maxillofacial surgeon on patients under local anesthesia (Articaine in 4% solution with 1:200,000 epinephrine; Ultracaine DS^®^, Aventis Parma, Istanbul, Turkey) and sterile conditions. The osteotomy (and crown sectioning in necessary cases) was performed using low-speed hand pieces (NSK, Tochigi, Japan) under sterile saline irrigation. Subsequently, curettage of the socket was performed and irregular bone borders were evened. Primary wound closure was performed using polyglactin resorbable sutures (3-0 Vicryl, Ethicon^®^, Cornelia, GA, USA). After surgery, all patients were prescribed antibiotics (Amoxicillin + clavulanate, Augmentin, Glaxo Smith Kline, Istanbul, Turkey), analgesics (Flurbiprofen, Majezik, Sanovel, Istanbul, Turkey), and antiseptic mouthwash (Benzydamine HCl + chlorhexidine gluconate, Farhex, Santa Farma, Istanbul, Turkey) for postoperative 7 days. The patients were informed to use the analgesics only when needed and record the number of pills used.

### Kinesio Taping

**Figure 1:**
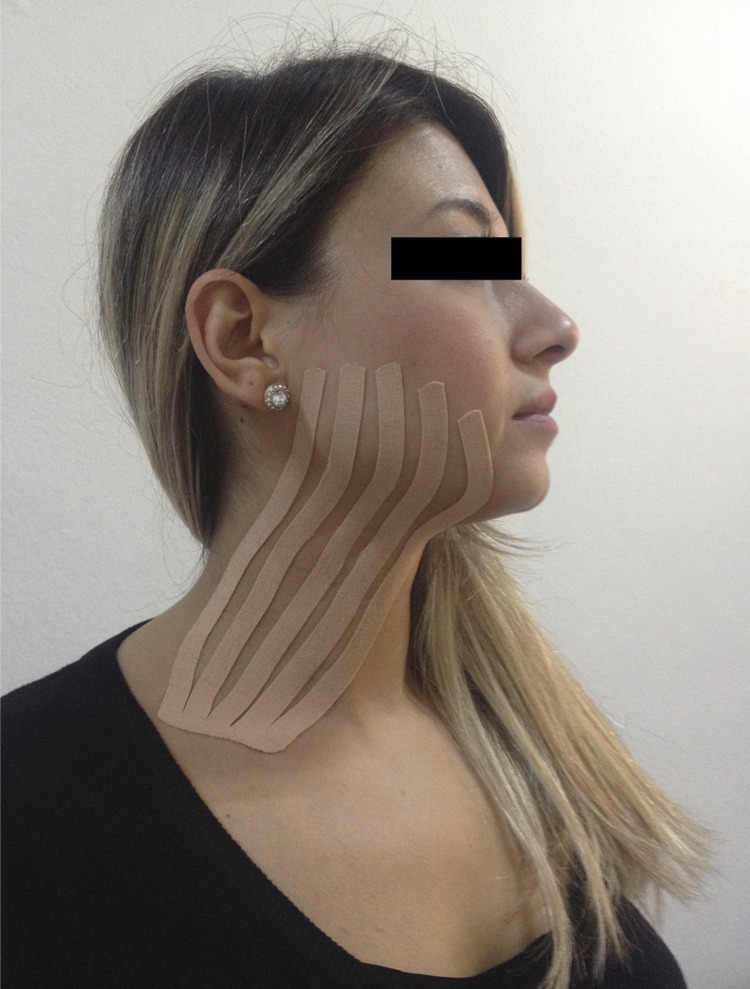
Clinical application of kinesio taping

Kinesio taping was performed just after the surgery. All taping procedures were performed by the same KT certified physician according to the lymphatic correction technique described by Kase, et al.^10^(2003) Kinesio^®^ Tex Gold^TM^was used as KT material. Before the application, patient’s skin was cleaned, and moisture- and oil-free condition was provided. The lymphatic correction technique was used to remove the edema. The taping material was cut into five pieces equal in width, leaving 1.5-2.0 cm uncut tape at the base. The base of the five-strip taping material was applied slightly above the supraclavicular lymph node without tension. The strips were then directed at the lymphatic duct and applied one by one with slight tension (15% of available) (Figure 1). One I-shaped kinesio strip was used for placebo taping. The strip was applied parallel to the axis of the corpus mandible. No tension was applied to the taping material. Tapes remained for 5 days.

### Data Collection

All measurements were performed by the same researcher. The surgery time was recorded for each patient as the time elapsed between initial incision and final suturing. Measurements and data collections were performed at four specific time points: baseline, 2^nd^, 4^th^, and 7^th^ postoperative days.

To evaluate trismus, maximum mouth opening (MMO) was recorded using calipers. The MMO was measured between the edges of upper and lower central incisors in millimeters.

Facial swelling was assessed by a three-line measurement method using a flexible plastic tape measure. The corresponding lines were tragus-ala nasi (Tr-An; from the most posterior point of the tragus to the most lateral point of the ala nasi), tragus-commissure (Tr-Co; from the most posterior point of the tragus to the most lateral point of the lip commissure), and tragus-pogonion (Tr-Po; from the most posterior point of the tragus to the pogonion) distances. These measurements were performed with the patient sitting at 90° straight position with physiologic rest position of the mandible.

The pain was evaluated by patient self-assessment using a visual analogue scale (VAS, 0-10 cm), in which the endpoints indicate “no pain” and “unbearable pain”. The patients were asked to put a mark along the VAS line to specify their pain experiences at two different states: VAS score for pain at resting (r-VAS) and chewing (c-VAS). The numbers of analgesic tablets taken were also recorded during follow-up visits.

### Statistical Analysis

The statistical analysis was performed using SPSS software (SPSS 17.0; Chicago, IL, USA). Descriptive analyses and chi-squared test were used to compare participants’ baseline characteristics. One-way ANOVA was used to test differences among the groups at the same time interval. Bonferroni-corrected post-hoc tests were used for multiple comparisons. The level of significance was set at p<0.05.

## Results

### Baseline Characteristics

In total, 60 patients that met the inclusion criteria were enrolled in the study and randomly allocated to one of the three study groups (20 patients in each). Our study was conducted without droppouts. The baseline characteristics of the groups, including age, gender, and operation time, are shown in Table 1. There were no differences among the groups regarding of baseline characteristics (p>0.05; the exact p-values are shown in Table 1). No complications were evident during study.

**Table 1:** Participants' baseline characteristics

Baseline characteristics	Groups			p
	Kinesio (n=20)	Placebo (n=20)	Control (n=20)	
Age, years, mean ± SD	27.2 ± 5.8	24.6 ± 5.2	25.3 ± 3.9	0.257^a^
Gender, female, n (%)	15 (75.0)	18 (90.0)	17 (85.0)	0.431^b^
Operation time, min, mean ± SD	25.8 ± 3.1	27.1 ± 2.7	26.8 ± 2.8	0.980^a^

^a^p-value for the comparison of age and operation time values among groups (one-way ANOVA)

^b^p-value for the comparison of gender distribution among groups (x2 test)

### Comparison of Facial Swelling Measurements

**Figure 2:**
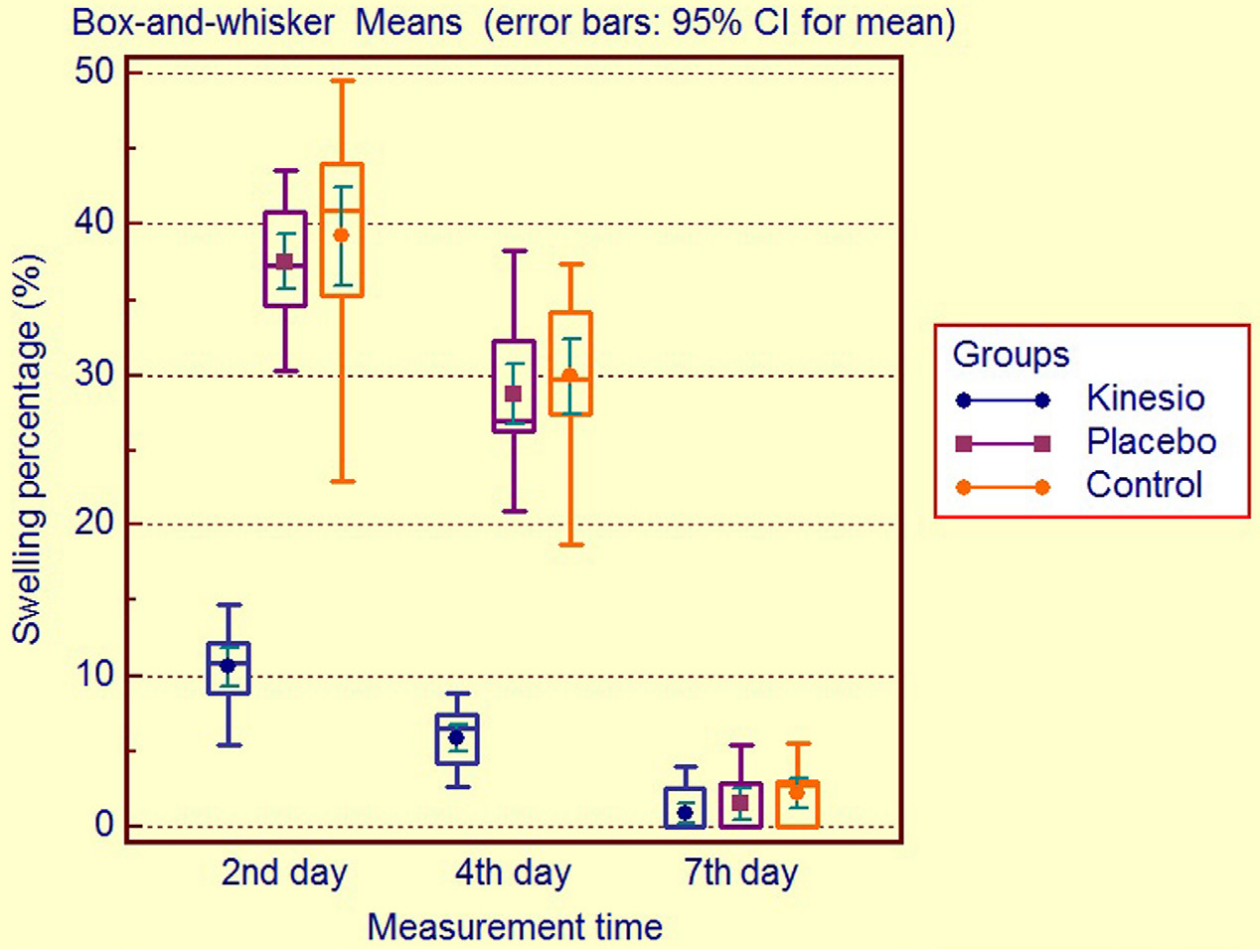
Box-and-whisker graphic of facial swelling percentage values of the patients of kinesio, placebo and control groups during follow-up visits

Facial swelling measurements were compared as both separate values of each three lines and as mean sum of all line values. The baseline facial measurements among the three groups were comparable (p>0.05). During the postoperative 2^nd^ and 4^th^ days, significant differences were observed among the groups (all p<0.001; the exact p-values were shown in Table 2). The mean sum of facial swelling values in placebo group was significantly lower than control group during the postoperative 2^nd^ and 4^th^ days (p=0.002 and p=0.006 respectively). Values in KT group were significantly lower than the other groups at both follow-up visits. During the postoperative 7^th^ day visit, all groups showed comparable swelling values (p>0.05). Facial swelling percentage values were also estimated using mean sum of the all three line measurements compared to baseline values (Figure 2). Regarding facial swelling percentages, KT group showed significantly lower values (10.6±2.7%) when compared with the placebo and control groups (37.5±3.9% and 39.2±7.0%, respectively) during the postoperative 2^nd^ day follow-up visit (all p<٠.٠٠١). Likewise, patients in KT group showed significantly less facial swelling values (5.9±1.9%) when compared with those in placebo and control groups (28.8±4.3% and 29.9±5.3%, respectively) on the 4^th^ day follow-up visit (all p<٠.٠٠١). However, placebo and control groups showed similar results on the 2^nd^ and 4^th^ day follow-up visits (p=0.823 and p=1.000, respectively). Comparable swelling percentage values were observed in all groups (0.9±1.4%, 1.5±2.2%, and 2.2±2.1%, respectively) on the 7^th^ postoperative day (p=0.112) (Figure 2).

**Table 2:** Comparison of facial swelling measurements (cm) among the three groups. All values are shown as mean ± SD

Swelling	Groups			p*	p1-2	p1-3	p2-3
	Kinesio (n=20)	Placebo (n=20)	Control (n=20)				
Baseline							
Tr-An	11.1 ± 0.9	10.5 ± 0.8	10.6 ± 1.0	0.215	0.295	0.552	1.000
Tr-Co	10.6 ± 0.9	10.7 ± 0.5	11.1 ± 0.8	0.144	1.000	0.218	0.329
Tr-Pg	15.0 ± 1.5	14.8 ± 0.4	14.8 ± 1.2	0.846	1.000	1.000	1.000
Sum	36.7 ± 2.9	36.1 ± 1.4	36.2 ± 2.7	0.728	1.000	1.000	1.000
2^nd^ postoperative day							
Tr-An	11.5 ± 0.9	13.1 ± 0.9	14.9 ± 1.2	<0.001	<0.001	<0.001	<0.001
Tr-Co	12.3 ± 0.9	15.8 ± 0.9	16.4 ± 1.1	<0.001	<0.001	<0.001	0.250
Tr-Pg	16.8 ± 1.6	19.6 ± 0.9	20.4 ± 1.4	<0.001	<0.001	<0.001	0.202
Sum	40.6 ± 3.0	48.5 ± 2.0	51.7 ± 3.3	<0.001	<0.001	<0.001	0.002
4^th^ postoperative day							
Tr-An	11.1 ± 0.9	12.3 ± 0.8	14.1 ± 1.3	<0.001	0.001	<0.001	<0.001
Tr-Co	10.8 ± 1.1	15.0 ± 0.9	15.2 ± 1.0	<0.001	<0.001	<0.001	1.000
Tr-Pg	15.1 ± 1.8	19.1 ± 1.2	19.3 ± 1.3	<0.001	<0.001	<0.001	1.000
Sum	37.1 ± 3.3	45.6 ± 1.8	48.6 ± 3.4	<0.001	<0.001	<0.001	0.006
7^th^ postoperative day							
Tr-An	11.0 ± 1.0	10.5 ± 0.8	10.6 ± 0.9	0.179	0.278	0.388	1.000
Tr-Co	10.6 ± 0.9	10.8 ± 0.6	11.2 ± 0.9	0.109	1.000	0.115	0.547
Tr-Pg	15.0 ± 1.5	15.2 ± 0.8	15.4 ± 1.1	0.492	1.000	0.710	1.000
Sum	35.7 ± 5.9	36.6 ± 1.8	37.1 ± 2.8	0.535	1.000	0.834	1.000

p*, p-value for the comparison among groups (one-way ANOVA)

p1-2, p-value for multiple comparison of kinesio and placebo groups (Bonferroni-corrected post-hoc test)

p1-3, p-value for multiple comparison of kinesio and control groups (Bonferroni-corrected post-hoc test)

p2-3, p-value for multiple comparison of placebo and control groups (Bonferroni-corrected post-hoc test)

### Comparison of Pain Measurements

During the postoperative 2^nd^ and 4^th^ day follow-up visits, significant differences were observed in pain VAS scores among the groups (Table 3, all p<0.001). After multiple comparisons, we found that pain values in KT group were significantly lower than in placebo and control groups on the postoperative 2^nd^ and 4^th^ days (the exact p-values are shown in Table 3). During the postoperative 7^th^ day follow-up visit, all groups showed comparable pain values in both resting and chewing states (Table 3). The patients of KT group took significantly less analgesic tablets than patients of placebo and control groups at all follow-up visits (Table 3). Regarding pain VAS scores and number of analgesic tablets, placebo and control groups revealed comparable outcomes in all follow-up visits.

**Table 3:** Comparison of pain parameters (r-VAS and c-VAS values, and total number of analgesic tablets taken) among the three groups. All values are shown as mean ± SD

Pain	Groups			p*	p1-2	p1-3	p2-3
	Kinesio (n=20)	Placebo (n=20)	Control (n=20)				
2^nd^ postoperative day							
r-VAS	1.9 ± 1.5	4.7 ± 3.0	4.4 ± 1.5	<0.001	<0.001	0.002	1.000
c-VAS	3.4 ± 1.7	5.8 ± 2.5	6.3 ± 1.5	<0.001	0.001	<0.001	1.000
Analgesic	2.9 ± 1.1	4.6 ± 1.1	5.3 ± 1.1	<0.001	<0.001	<0.001	0.205
4^th^ postoperative day							
r-VAS	0.6 ± 0.9	2.1 ± 1.7	2.5 ± 1.3	<0.001	0.003	<0.001	1.000
c-VAS	1.2 ± 1.3	3.5 ± 1.9	4.4 ± 1.5	<0.001	<0.001	<0.001	0.294
Analgesic	4.5 ± 2.2	7.1 ± 1.2	7.9 ± 1.7	<0.001	<0.001	<0.001	0.329
7^th^ postoperative day							
r-VAS	0.1 ± 0.3	0.1 ± 0.2	0.1 ± 0.3	0.812	1.000	1.000	1.000
c-VAS	0.2 ± 0.4	0.2 ± 0.5	0.4 ± 0.7	0.503	1.000	0.785	1.000
Analgesic	5.1 ± 2.1	8.7 ± 1.2	9.3 ± 1.4	<0.001	<0.001	<0.001	0.878

r-VAS, visual analog score at resting state; c-VAS, visual analog score at chewing state.

For description of p-values, please see footnote of Table 2.

### Comparison of Trismus Measurements

The baseline MMO values were comparable in the three groups (p=0.780) (Table 4). We found that MMO values in KT group were significantly higher than in placebo and control groups on the 2^nd^ and 4^th^ day follow-up visits (The exact p-values are shown in Table 4). However, all groups showed comparable MMO values during postoperative 7^th^ day follow-up visit (p=0.205). Considering pairwise comparison of MMO, placebo and control groups showed comparable results in all follow-up visits (Table 4).

**Table 4:** Comparison of mouth-opening values (mm) among the patients of the three groups

Group	n		Mouth Opening (mean ± SD)		
		Baseline	2^**nd**^ day	4^**th**^ day	7^**th**^ day
Kinesio	20	45.7 ± 3.8	34.5 ± 9.8	42.8 ± 6.6	44.1 ± 4.2
Placebo	20	45.5 ± 4.6	27.7 ± 2.6	30.0 ± 3.3	43.4 ± 4.8
Control	20	44.7 ± 5.1	24.0 ± 3.7	29.2 ± 2.8	41.9 ± 2.6
p*		0.780	<0.001	<0.001	0.205
p1-2		1.000	0.003	<0.001	1.000
p1-3		1.000	<0.001	<0.001	0.253
p2-3		1.000	0.210	1.000	0.663

For description of p-values, please see footnote of Table 2.

## Discussion

Postoperative swelling, pain, and limitation of mouth opening are common after impacted third molar surgeries and severely decrease the patients’ quality of life. Despite the increased clinical application of KT in physiotherapy practice, especially after traumatic injuries, only a few studies investigate the effectiveness of KT in OMS literature; two studies showing effects of KT after maxillofacial trauma,^17,19^ four studies after impacted tooth surgery,^5,15,16,18^ two studies after orthognathic surgery,^20,21^ and one study for temporomandibular joint disorders.^23^ However, the aforementioned studies did not include placebo taping. Thus, the actual efficacy of KT application, distinguished from the placebo effects, on postoperative morbidity after OMS is not clear in the literature.

Ristow, et al.^19^ (2014) revealed that KT application significantly reduced swelling but were not effective against pain and trismus control after surgical treatment of zygomatico-orbital fractures. In another study, Ristow, et al.^17^ (2013) reported that KT did not significantly reduce postoperative trismus and pain after surgical treatment of mandibular fractures. Likewise, Tozzi, et al.^20^ (2016) reported that KT had a significant effect on reduction of facial swelling but no effect on pain and trismus reduction after orthognathic surgery. These studies did not contain placebo group. Thus, the aforementioned insignificant effects of KT on pain and trismus control might be due to the placebo effects pooled in the kinesio group. Ristow, et al.^18^ (2014) showed that KT resulted in significantly lower postoperative swelling, pain and trismus on day 2 and 3 after impacted third molar surgery when compared with the control group. In a recent split-mouth clinical study, da Rocha Heras, et al.^15^ (2020) concluded that KT was effective in reducing edema and pain after impacted third molar surgery. A recent split-mouth clinical study by Gözlüklü, et al.^16^ (2020) reported that their newly described KT method was more effective when compared with classic KT method in reducing postoperative morbidity after impacted third molar surgery. Genc, et al.^5^ (2019) compared the effectiveness of KT and surgical drain on postoperative morbidity after third molar surgery, observing significantly greater swelling and pain in KT group, and comparable occurrence of trismus in both groups. Lietz-Kijak, et al.^21^ (2018) revealed that KT had beneficial effects on the reduction of facial swelling after orthognathic surgery. Coskun Benlidayi, et al.^23^ (2016) investigated the efficacy of KT in patients with TMD regarding functional jaw movements, pain scores for joint and masticatory muscles, and depression and disability scores. The authors reported that additional KT application was more effective for treatment of TMDs than counseling and exercise alone. Another study reported that KT application on latent myofascial trigger points significantly decreased the pain scores and increased the range of motion of TMJ.^24^ A recent study by Keskinruzgar, et al.^25^ (2019) compared the efficacy of KT and occlusal splint therapy for management of myofascial pain in patients with sleep bruxism. The authors concluded that KT was an effective method in reducing muscle pain and increasing mouth opening, also showing comparable results with splint therapy. However, the aforementioned studies did not contain placebo group, which might bring into question KT real effectiveness.

In our study, the patients were divided into three groups: kinesio, placebo, and control. One of the novel features was that placebo effect of taping was also compared with kinesio and control groups. To the best our knowledge, this is the first study showing the actual effectiveness of KT therapy on recovery after impacted mandibular third molar surgery that included placebo-controlled results. This special emphasis on placebo taping might allow us to differentiate the true merit of kinesio taping after surgery-related trauma in maxillofacial region. The patients with solely mandibular impacted tooth with similar impact level were included in our study to provide standardization. Furthermore, all surgeries were performed by the same oral surgeon in comparable operation time and all KT applications were performed by same professional. Patients’ baseline characteristics in all groups were comparable. Thus, the differences among the groups can be attributed to the efficacy of KT application.

Our study showed that KT application significantly reduced postoperative morbidity (swelling, pain and trismus) on days 2 and 4 after impacted tooth surgery. The effectiveness of KT ended on 7^th^ postoperative day. Our study also contained placebo and control groups that did not show similar significant effects when compared with the kinesio group. Thus, we can conclude with confidence that our hypothesis was mostly confirmed.

Some factors affect the effectiveness of KT, such as tape thickness, adhesion and stretch capacity, and correct application technique.^12^ The KT can stretch up to 1.4 fold of its original length, and recoils back to its original length during the following days. For proper KT application, the head of the patient should be rotated and the muscles should be extended in order to stretch the skin before taping. When head of the patient returns to its resting position, the elastic band subsequently recoils back and forms convolutions on the taped skin. When the taping technique is correctly applied, the tape pulls the skin and increases the interstitial space between the skin and connective tissue, thus promoting the hemorrhagic and lymphatic drainage.^12^ Since KT is thought to improve the blood and lymph flow, it has become a popular method in the management of lymphedema.^11^ In our study, we observed that in addition to decreasing swelling, KT reduced trismus and pain, possibly due to decreased lymphedema and skin tension. However, these effects were not seen in placebo group (taping without tension). Considering favorable effects of KT on pain and trismus, our results were different from previous studies,^17,19,20^ in which KT was reported to be ineffective on pain and trismus control. In consistence with previous investigations,^17-19^ the KT was removed on the 5^th^ postoperative day in our study. However, Genc, et al.^5^ (2019) reported that the tape was removed on the 2^nd^ postoperative day. The application time of the therapeutic tape might also influence KT effectiveness.

In our study, the methods for swelling, pain and trismus measurement were precise, simple, inexpensive, and of easy approach, also being reported in many previous studies.^1,5,15,17-19,21^ The 3-D assessment of postoperative facial swelling was reported as a novel and accurate method in recent studies.^8,16,20,26^ However, this method has not yet been considered easy, practical, cheap, and widely available. One of the novel aspects of our study was that the effectiveness of KT on facial swelling was reported by each line measurement separately as well as mean sum values. In all of the previous studies,^5,17-19,21^ facial swelling was reported only by mean sum of all line measurements. Due to this separate reporting emphasis, effectiveness of KT on each line region could be investigated in detail. Our study revealed that placebo taping showed significantly less facial swelling values on the Tr-An line but comparable swelling values on the Tr-Co and Tr-Pg lines on the 2^nd^ and 4^th^ days of follow-up visits. However, KT application significantly decreased swelling in all line measurements.

Our research showed that approximately 10.6% increase in mean facial swelling was observed in patients in kinesio group on the 4^th^ postoperative day, whereas the patients in placebo and control groups showed about 37.5% and 39.2% increase, that is, approximately 3.6 fold more swelling. Considering trismus, the mean MMO values of the patients in kinesio group reached above 40mm on the 4^th^ postoperative day, that is, no limitation at mouth opening, whereas the mean MMO values in placebo and control groups were below 35 mm, showing ongoing trismus. These results mean that patients in kinesio group might go back to their social and professional life earlier compared to the placebo and control groups. Previous studies reported that kinesio caused early peak swelling and faster swelling reduction after surgery.^18,19^ Thus, KT application may decrease the workforce loss of the individuals and may have positive influence on economic impact. Moreover, the patients treated with kinesio therapy needed significantly less analgesic drugs, possibly reducing and/or anticipating drug-related side-effects.

One of the limitations of our study was that it was not a split-mouth study. Different patients were assigned into three different study groups. A split-mouth study design might provide better evidence level. However, patients’ baseline characteristics, including age, gender, and operation time distribution, showed comparable results. Another limitation might be that swelling was not assessed with 3D methods, which could provide volume comparison. However, tape measuring method used to assess swelling in our study was frequently used in previous studies and reported to be reliable.^5,15,17-19,21^ Thus, we could compare our results with previous similar studies.

Although absent in our study, taping-related complications such as irritation or allergic reactions on skin should be considered. Thus, patients should be informed about possible adverse reactions before taping, especially those with sensitive skin.

## Conclusion

The KT application is an effective method for reducing postoperative morbidity (swelling, pain, and trismus) after impacted mandibular third molar surgery. However, placebo taping is not as effective as proper taping. Placebo taping shows similar results compared to no taping regarding facial swelling percentage, pain, and trismus.

## References

[1] - Giovannacci I, Giunta G, Pedrazzi G, Meleti M, Manfredi M, Migliario M, et al. Erbium Yttrium-Aluminum-Garnet laser versus traditional bur in the extraction of impacted mandibular third molars: Analysis of intra- and postoperative differences. J Craniofac Surg. 2018;29(8):2282-6. doi: 10.1097/SCS.0000000000004574 10.1097/SCS.0000000000004574 29742567

[2] - Al-Khateeb TH, Nusair Y. Effect of the proteolytic enzyme serrapeptase on swelling, pain and trismus after surgical extraction of mandibular third molars. Int J Oral Maxillofac Surg. 2008;37(3):264-8. doi: 10.1016/j.ijom.2007.11.011 10.1016/j.ijom.2007.11.011 18272344

[3] - Kim K, Brar P, Jakubowski J, Kaltman S, Lopez E. The use of corticosteroids and nonsteroidal antiinflammatory medication for the management of pain and inflammation after third molar surgery: a review of the literature. Oral Surg Oral Med Oral Pathol Oral Radiol Endod. 2009;107(5):630-40. doi: 10.1016/j.tripleo.2008.11.005 10.1016/j.tripleo.2008.11.005 19157919

[4] - Koyuncu BO, Zeytinoglu M, Tetik A, Gomel MM. Effect of tube drainage compared with conventional suturing on postoperative discomfort after extraction of impacted mandibular third molars. Br J Oral Maxillofac Surg. 2015;53(1):63-7. doi: 10.1016/j.bjoms.2014.09.021 10.1016/j.bjoms.2014.09.021 25451073

[5] - Genc A, Cakarer S, Yalcin BK, Kilic BB, Isler SC, Keskin C. A comparative study of surgical drain placement and the use of kinesiologic tape to reduce postoperative morbidity after third molar surgery. Clin Oral Investig. 2019;23(1):345-50. doi: 10.1007/s00784-018-2442-x 10.1007/s00784-018-2442-x 29675759

[6] - Eshghpour M, Ahrari F, Takallu M. Is low-level laser therapy effective in the management of pain and swelling after mandibular third molar surgery? J Oral Maxillofac Surg. 2016;74(7):1322.e1-8. doi: 10.1016/j.joms.2016.02.030 10.1016/j.joms.2016.02.030 27055228

[7] - Metin R, Tatli U, Evlice B. Effects of low-level laser therapy on soft and hard tissue healing after endodontic surgery. Lasers Med Sci. 2018;33(8):1699-706. doi: 10.1007/s10103-018-2523-8 10.1007/s10103-018-2523-8 29713842

[8] - Rana M, Gellrich NC, Ghassemi A, Gerressen M, Riediger D, Modabber A. Three-dimensional evaluation of postoperative swelling after third molar surgery using 2 different cooling therapy methods: A randomized observer-blind prospective study. J Oral Maxillofac Surg. 2011;69(8):2092-8. doi: 10.1016/j.joms.2010.12.038 10.1016/j.joms.2010.12.038 21496998

[9] - Szolnoky G, Szendi-Horváth K, Seres L, Boda K, Kemény L. Manual lymph drainage efficiently reduces postoperative facial swelling and discomfort after removal of impacted third molars. Lymphology. 2007;40(3):138-42. 18062616

[10] - Kase K, Wallis J, Kase T. Clinical Therapeutic Applications of the Kinesio Taping Method. 2nd Ed. Tokyo: Ken Ikai Co Ltd; 2003.

[11] - Kase K, Stockheimer KR. Kinesio taping for lymphoedema and chronic swelling - Kinesio Taping Manual. Tokyo: Ken Ikai Co Ltd; 2006.

[12] - Sijmonsma J. Lymph taping: theory, technique, practice. Goor: Fysionair VOF; 2010.

[13] - Williams S, Whatman C, Hume PA, Sheerin K. Kinesio taping in treatment and prevention of sports injuries: a meta-analysis of the evidence for its effectiveness. Sports Med. 2012;42(2):153-64. doi: 10.2165/11594960-000000000-00000 10.2165/11594960-000000000-00000 22124445

[14] - Ghozy S, Dung NM, Morra ME, Morsy S, Elsayed GG, Tran L, et al. Efficacy of kinesio taping in treatment of shoulder pain and disability: a systematic review and meta-analysis of randomised controlled trials. Physiotherapy. 2020;107:176-88. doi: 10.1016/j.physio.2019.12.001 10.1016/j.physio.2019.12.001 32026818

[15] - Rocha Heras AC, Oliveira DM, Guskuma MH, Araújo MC, Fernandes KB, Silva RA Junior, et al. Kinesio taping use to reduce pain and edema after third molar extraction surgery: A randomized controlled split-mouth study. J Craniomaxillofac Surg. 2020;48(2):127-31. doi: 10.1016/j.jcms.2019.12.003 10.1016/j.jcms.2019.12.003 31899111

[16] - Gözlüklü Ö, Ulu M, Gözlüklü HÖ, Yilmaz N. Comparison of different kinesio taping techniques after third molar surgery. J Oral Maxillofac Surg. 2020;78(5):695-704. doi: 10.1016/j.joms.2019.12.026 10.1016/j.joms.2019.12.026 32008990

[17] - Ristow O, Hohlweg-Majert B, Kehl V, Koerdt S, Hahnefeld L, Pautke C. Does elastic therapeutic tape reduce postoperative swelling, pain, and trismus after open reduction and internal fixation of mandibular fractures? J Oral Maxillofac Surg. 2013;71(8):1387-96. doi: 10.1016/j.joms.2013.03.020 10.1016/j.joms.2013.03.020 23676774

[18] - Ristow O, Hohlweg-Majert B, Stürzenbaum SR, Kehl V, Koerdt S, Hahnefeld L, et al. Therapeutic elastic tape reduces morbidity after wisdom teeth removal: a clinical trial. Clin Oral Investig. 2014;18(4):1205-12. doi: 10.1007/s00784-013-1067-3 10.1007/s00784-013-1067-3 23963616

[19] - Ristow O, Pautke C, Victoria Kehl, Koerdt S, Schwärzler K, Hahnefeld L, et al. Influence of kinesiologic tape on postoperative swelling, pain and trismus after zygomatico-orbital fractures. J Craniomaxillofac Surg. 2014;42(5):469-76. doi: 10.1016/j.jcms.2013.05.043 10.1016/j.jcms.2013.05.043 23830769

[20] - Tozzi U, Santagata M, Sellitto A, Tartaro GP. Influence of kinesiologic tape on post-operative swelling after orthognathic surgery. J Maxillofac Oral Surg. 2016;15(1):52-8. doi: 10.1007/s12663-015-0787-0 10.1007/s12663-015-0787-0 PMC4759034 26929553

[21] - Lietz-Kijak D, Kijak E, Krajczy M, Bogacz K, Łuniewski J, Szczegielniak J. The impact of the use of kinesio taping method on the reduction of swelling in patients after orthognathic surgery: a pilot study. Med Sci Monit. 2018;24:3736-43. doi: 10.12659/MSM.909915 10.12659/MSM.909915 PMC6015478 29861496

[22] - Pell GJ, Gregory GT. Report on a ten-year study of a tooth division technique for the removal of impacted teeth. Am J Orthod Oral Surg. 1942;28:B660-6.

[23] - Coskun Benlidayi I, Salimov F, Kurkcu M, Guzel R. Kinesio taping for temporomandibular disorders: single-blind, randomized, controlled trial of effectiveness. J Back Musculoskelet Rehabil. 2016;29(2):373-80. doi: 10.3233/BMR-160683 10.3233/BMR-160683 26966829

[24] - Bae Y. Change the myofascial pain and range of motion of the temporomandibular joint following kinesio taping of latent myofascial trigger points in the sternocleidomastoid muscle. J Phys Ther Sci. 2014;26(9):1321-4. doi: 10.1589/jpts.26.1321 10.1589/jpts.26.1321 PMC4175229 25276008

[25] - Keskinruzgar A, Kucuk AO, Yavuz GY, Koparal M, Caliskan ZG, Utkun M. Comparison of kinesio taping and occlusal splint in the management of myofascial pain in patients with sleep bruxism. J Back Musculoskelet Rehabil. 2019;32(1):1-6. doi: 10.3233/BMR-181329 10.3233/BMR-181329 30475753

[26] - Rana M, Gellrich NC, von See C, Weiskopf C, Gerressen M, Ghassemi A, et al. 3D evaluation of postoperative swelling in treatment of bilateral mandibular fractures using 2 different cooling therapy methods: a randomized observer blind prospective study. J Craniomaxillofac Surg. 2013;41(1):e17-23. doi: 10.1016/j.jcms.2012.04.002 10.1016/j.jcms.2012.04.002 22626630

